# Low genetic heterogeneity of copy number variations (CNVs) in the genes encoding the human deoxyribonucleases 1-like 3 and II potentially relevant to autoimmunity

**DOI:** 10.1371/journal.pone.0215479

**Published:** 2019-04-25

**Authors:** Misuzu Ueki, Junko Fujihara, Kaori Kimura-Kataoka, Kazuo Yamada, Yoshikazu Takinami, Haruo Takeshita, Reiko Iida, Toshihiro Yasuda

**Affiliations:** 1 Division of Medical Genetics and Biochemistry, School of Medical Sciences, University of Fukui, Eiheiji, Fukui, Japan; 2 Department of Legal Medicine, Shimane University School of Medicine, Izumo, Shimane, Japan; 3 Department of Emergency and Critical Care Medicine, Shimane University School of Medicine, Izumo, Shimane, Japan; 4 Division of Life Science, School of Medical Sciences, University of Fukui, Eiheiji, Fukui, Japan; Marquette University, UNITED STATES

## Abstract

Deoxyribonucleases (DNases) might play a role in prevention of autoimmune conditions such as systemic lupus erythematosus through clearance of cell debris resulting from apoptosis and/or necrosis. Previous studies have suggested that variations in the *in vivo* activities of DNases I-like 3(1L3) and II have an impact on autoimmune-related conditions. The genes for these DNases are known to show copy number variations (CNVs) whereby copy loss leads to a reduction of the *in vivo* activities of the enzymes, thereby possibly affecting the pathophysiological background of autoimmune diseases. Using a simple newly developed quantitative real-time PCR method, we investigated the distributions of the CNVs for *DNASE1L3* and *DNASE2* in Japanese and German populations. It was found that only 2 diploid copy numbers for all of these *DNASE* CNVs was distributed in both of the study populations; no copy loss or gain was evident for any of the autoimmune-related DNase genes. Therefore, it was demonstrated that these human autoimmune-related DNase genes show low genetic diversity of CNVs resulting in alterations of the *in vivo* levels of DNase activity.

## Introduction

It has been suggested that deoxyribonuclease (DNase)-mediated clearance of cell debris resulting from apoptosis and/or necrosis might be primarily involved in the prevention of autoimmune conditions such as systemic lupus erythematosus (SLE) [1,2]. In the context of autoimmunity, it has been postulated that DNase I-like 3 (DNase 1L3) in serum break down chromatin during apoptosis and/or necrosis [3,4], while DNase II in lysosomes is involved in the degradation of endogenous DNA in apoptotic cells that have been engulfed by macrophages [5,6].

A null variant resulting from a homozygous 1-bp deletion and homozygosity for a missense mutation (p.Arg206Cys), the latter resulting in an inactive form of the DNase 1L3 enzyme [7], have been found in an SLE patient [8]. Furthermore, in the patients with hypocomplementemic urticarial vasculitis syndrome being linked to SLE, a homozygous frameshift mutation, together with that leading to exon-skip were identified in *DNASE1L3* [9]. It has also been reported that DNase II–deficient mice develop a rheumatoid arthritis–like syndromes [10] and that lupus-prone MRL and NZB/W F1 mice have impaired DNase 1L3 activity[11]. These findings strongly suggest that reduction in the *in vivo* levels of these DNase activities might be substantially responsible for the genetic backgrounds determining susceptibility to autoimmune diseases.

**Fig 1:**
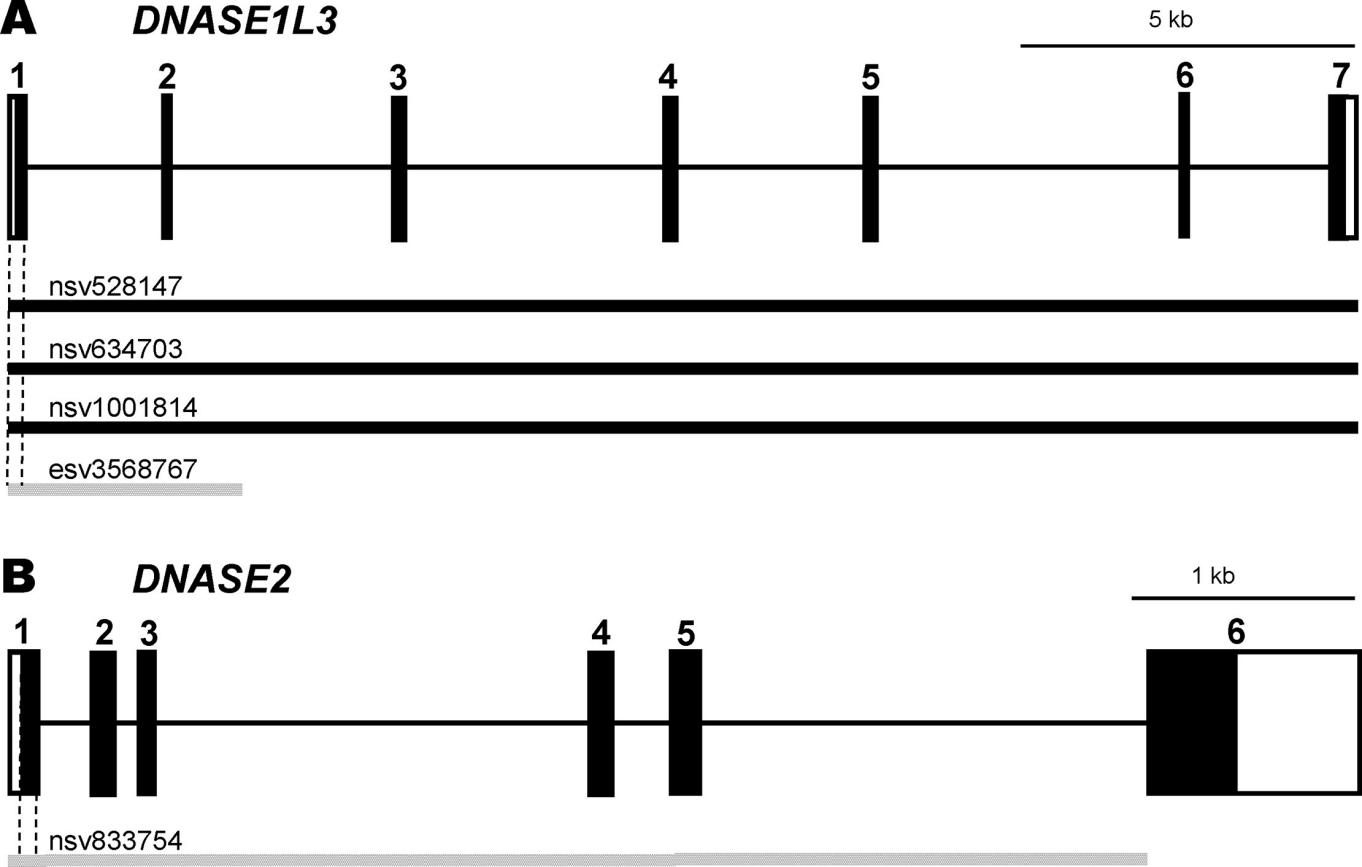
**The CNVs in (A) *DNASE1L3* and (B) *DNASE2*.** The genomic structures of each DNase gene were based upon the NCBI Reference Sequence: NG_032070.1 and NC_000019.10, respectively. All the CNVs in each gene were registered in the Database of Genomic Variants (http://dgv.tcag.ca/gb2/gbrowse/dgv2_hg38/). The black and gray bars indicate gain and loss of copy, respectively, in the CNVs. The regions of the gene shown by the dashed lines are the target regions used for Q-PCR analysis of each gene.

Variations in copy number, representing a type of genomic structural variation, are defined as segments of DNA 1 kb or longer in length than a reference genome [12,13]. Chromosomal regions affected by copy number variation (CNVs) are estimated to be much more numerous than those affected by nucleotide substitutions [14]. A significant proportion of CNVs are considered to affect gene function through dosage alteration, gene disruption, or a change in the level of expression. In the genes encoding the autoimmune-related DNases mentioned above, several CNVs including loss or gain of the copy have been characterized (Fig 1). It is plausible that loss of the copy in CNVs could give rise to a reduction in the *in vivo* level of each DNase activity, thereby affecting the etiology and pathophysiological background of autoimmune diseases. However, no genetic characterization of CNVs present in these DNase genes has been performed.

Recently, we have developed a simple quantitative real-time PCR (Q-PCR) method for screening of CNVs associated with physical features [15]. The strength of our method is that there is no requirement for obtaining reference DNA with a known copy number, whereas most other methods require reference DNA to determine and/or confirm the copy number by comparison between samples and reference DNA. Recently, we confirmed the reliability of the results obtained by our method using quantification of amplicons with an Agilent 2100 Bioanalyzer [16]. In the present study, the distributions of CNVs present in the genes encoding DNases 1L3 and II in Japanese and German populations were examined using the Q-PCR method, in which the common regions covering all of the CNVs leading to alterations in the *in vivo* activity levels of DNases 1L3 were chosen as targets for amplification.

## Materials and methods

Blood samples were collected from healthy unrelated Japanese (*n* = 265) and German (*n* = 80) individuals after obtaining written informed consent. DNA was extracted from each blood sample using a QIAamp DNA Mini Kit (Qiagen, Chatsworth, CA, USA). The study was approved by the Human Ethics Committee of Shimane University School of Medicine (the approval number 1024 for the Human Genome and Genetics Analysis Study).

We chose the common region of CNVs (nsv528147, nsv634703, nsv1001814 and esv3568767) in *DNASE1L3* and the target region of CNV (nsv833754) in *DNASE2* registered in the Database of Genomic Variants (http://dgv.tcag.ca/gb2/ gbrowse/ dgv2_hg38/). The *ZNF80* gene was chosen as a single-copy reference gene, according to a previous study using Q-PCR analysis [17]. All of the primers used in this study were designed using the NCBI Primer-BLAST software, except those for the *ZNF80* gene which were selected from the public RTPrimerDB database (RTPrimer DB ID: 1021), as shown in Table 1. Each of the gene-specific sequences was PCR-amplified using human genomic DNA as a template and the primer set DN1L3-S1/-AE, DN2-S1/-AE or ZNF80-SN/-A1, then cloned into the pCR II vector (Invitrogen, San Diego, CA, USA), being designated as pCRII/DN1L3, pCRII/DN2, and pCRII/ZNF, respectively. Next, the chimeric plasmid vectors containing both one copy of the DNase gene-specific sequence and the *ZNF80* gene-specific sequence in a tandem manner were prepared using the PCR gene fusion technique [18]; e.g., two PCR amplicons from *DNASE1L3* and *ZNF80* amplified separately were mixed with the primer set DN1L3-S1/ZNF-A1 and extended by PCR to form the chimeric sequence. The PCR-amplified product was then cloned into the pCRII vector, and designated as DN1L3-ZNF. The other chimeric vector DN2-ZNF was constructed in the same manner. All the constructs were confirmed by DNA sequence analysis and purified using the Plasmid Midi kit (Qiagen) for subsequent analysis.

**Table 1:** Primers† used in this study.

Gene	Primer	Orientation	Sequence (5’-3’) ‡	Positions§	Amplicon size (bp)	Targeted region
*DNASE1L3*	DN1L3-S1	forward	TCAGTGGAGCCTGCGGAAGT	11‒30	81	exon 1
	DN1L3-AE	reverse	AGCTGCAGGTCACAGCTTTC-			
			CGCTGCTCTGGCTTCAAGACT			
	DN1L3-A1	reverse	CGCTGCTCTGGCTTCAAGACT	72‒92		
*DNASE2*	DN2-S1	forward	CTGTACCCTCGTGATGTCCCC	5‒25	120	exon 1
	DN2-AE	reverse	AGCTGCAGGTCACAGCTTTC-			
			AGGAATCTGTGTCGGGGACTGC			
	DN2-A1	reverse	AGGAATCTGTGTCGGGGACTGC	104‒125		
*ZNF80*	ZNF80-S1	forward	CTGTGACCTGCAGCTCATCCT	1431‒1452	120	
	ZNF80-SN	forward	GAAAGCTGTGACCTGCAGCTCATCCT			
	ZNF80-A1	reverse	TAAGTTCTCTGACGTTGACTGATGTG-	1526‒1551		
			ATGTG			

†For the Q-PCR analysis, primer sets DN1L3-S1/ -A1, DN2-S1/ -A1, or ZNF80-S1/ -A1 were used, respectively.

‡Nucleotide tags for construction of chimeric plasmid DNAs are underlined.

§The positions of each primer are based on the genomic sequences of each DNases; DNase 1L3, NCBI Reference Sequence: NG_032070.1; DNase II, NCBI Reference Sequence: NC_000019.10; ZNF80, NCBI Reference Sequence: NC_000003.12.

Quantitative real-time PCR was performed with the StepOne Plus real-time PCR system (Applied Biosystems, Foster City, CA, USA). The important feature of this method is construction of a chimeric plasmid vector that includes one copy each of the single-copy (reference) gene-specific sequence and the target CNV-specific sequence, and its use for construction of standard curves for both the reference gene and the target CNV regions. Standard curves for the target CNV regions derived from each *DNase* and the *ZNF80* genes were separately constructed using a 2-fold dilution series of the corresponding chimeric vector preparation as a template. The specificity of the products amplified was evaluated by melting curve analysis. Q-PCR amplification for determination of the CNV copy number was performed in a total volume of 20 μl, containing 10 ng of genomic DNA, 2 pmol of the primer pairs as shown in Table 1 and 10 μl of Power SYBR Green Master Mix (Applied Biosystems). The cycling conditions were: 95°C for 10 min, followed by 40 cycles of 95°C for 15 s, 61°C for 1 min. For accuracy, Q-PCR samples for test and control constructs were run on a single plate at the same time. All of the PCR assays were performed in triplicate. Since two copies of the *ZNF80* gene are involved per diploid genome, the diploid copy number of the CNVs in each *DNase* gene was calculated using the formula: (amount of *DNASE* amplicons)/ (amount of *ZNF80* amplicons) x 2 The allowable error range for copy number estimation was set at ±0.4, as reported previously [15].

## Results

**Fig 2:**
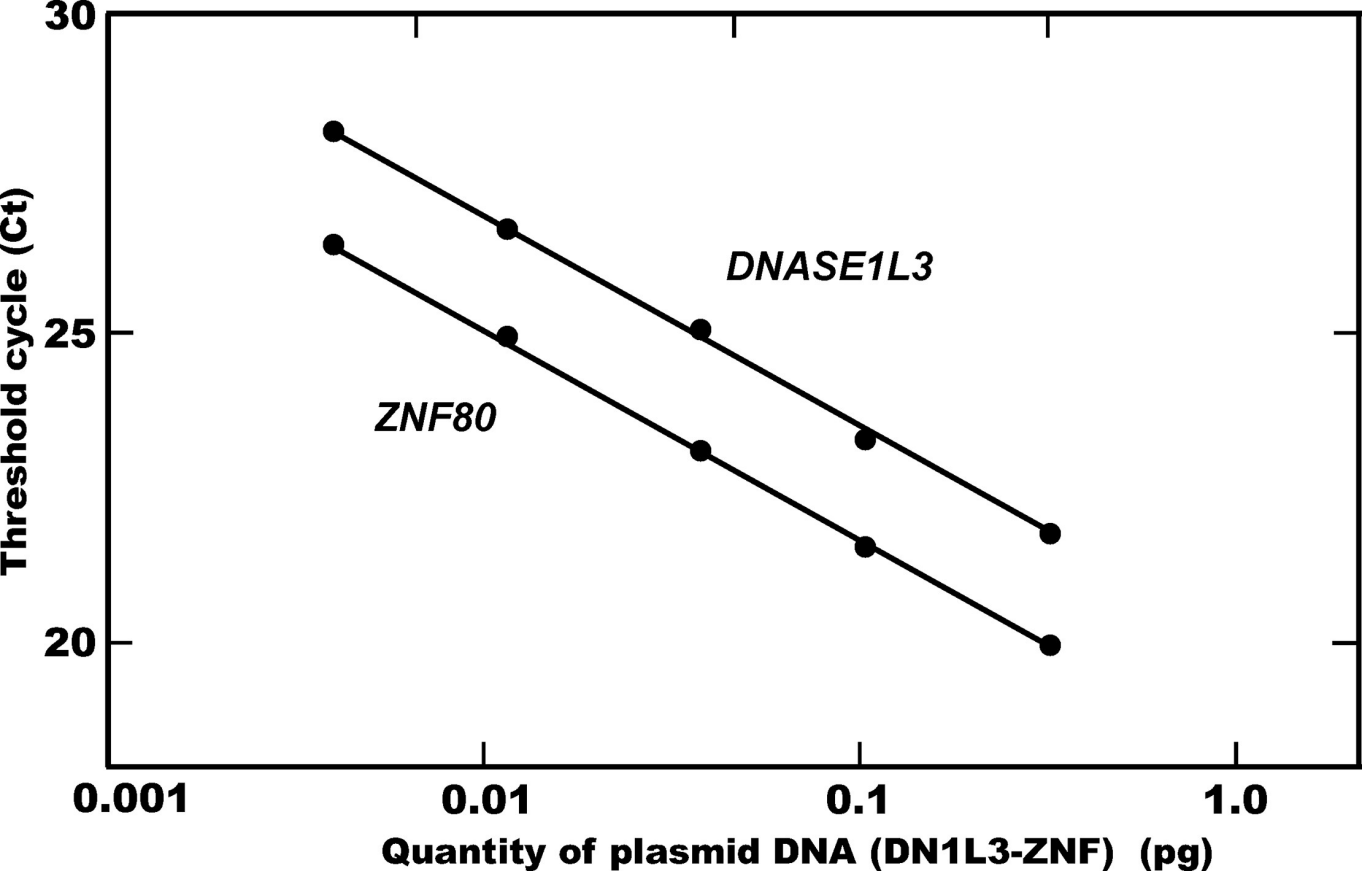
Standard curve of Q-PCR used for copy number analyses of the *DNASE1L3* CNV. The standard curves were constructed by plotting a known amount of the chimeric vector, DN1L3-ZNF, in serial 2-fold dilutions (0.014 to 0.23 pg) against the corresponding threshold cycles (Ct values) of the amplification plots. In the same manner, the standard curves for *DNASE2* CNV analysis were constructed using each of the chimeric vector DN2-ZNF.

**Fig 3:**
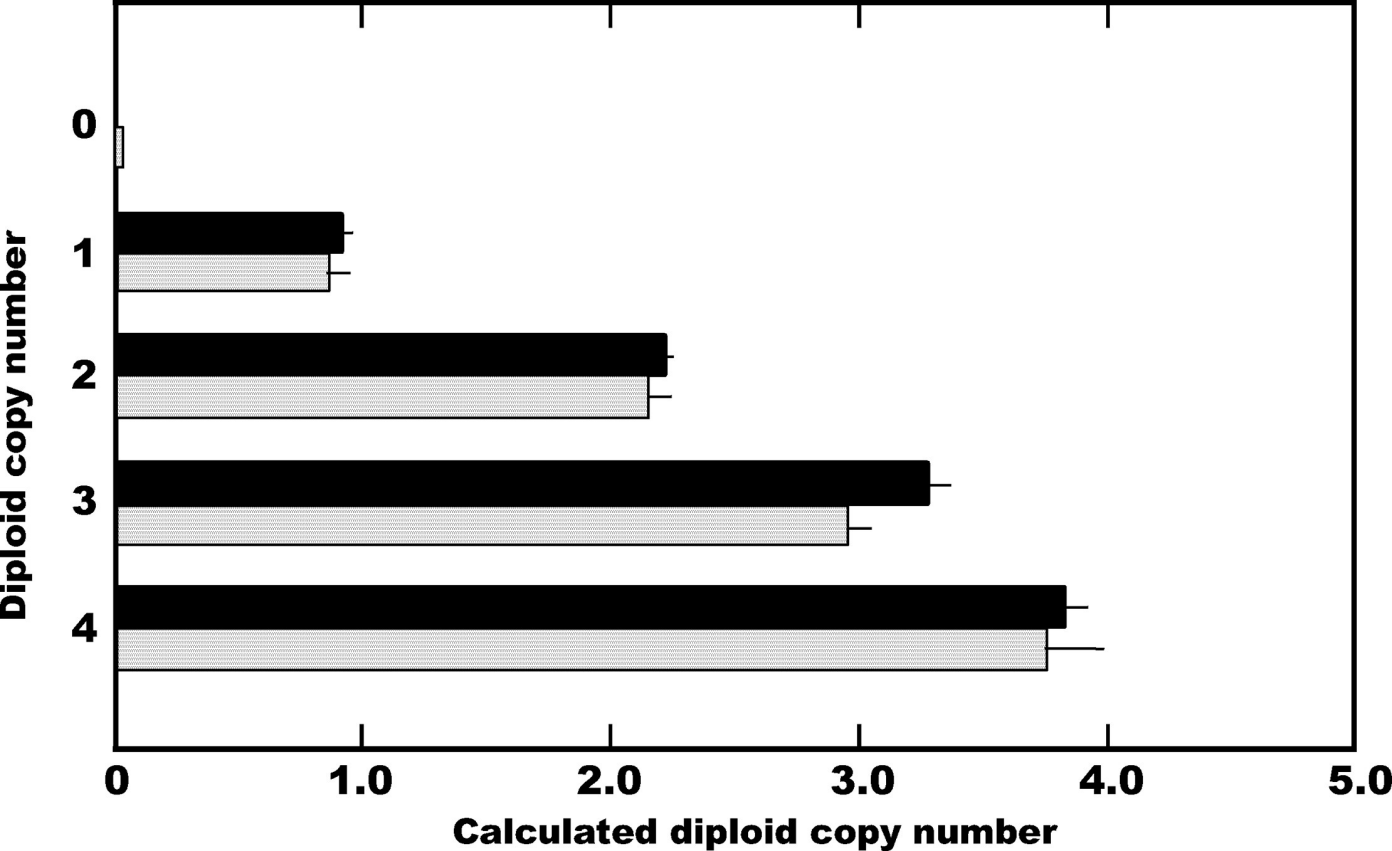
Simulated analysis of the *DNASE1L3* CNV copy number. Three vectors–pCRII/DN1L3, pCRII/ZNF and DN1L3-ZNF–were mixed in varying proportions to prepare mock DNA samples; for the expected diploid copy number 0, 1, 2, 3 and 4, these vectors were mixed at a molar ratio of 0:1:0, 0:1:1, 0:0:1, 1:0:2 and 1:0:1, respectively. Gray and black bars show the calculated diploid copy number when 0.04 and 0.11 pg of plasmid DNA in total were used as the template, respectively. The bars represent the mean ±S.D. of the results from three independent experiments.

We selected common region as a target CNV regions for amplification by Q-PCR analysis, covering all the CNVs in the gene encoding DNase 1L3 including loss or gain of copy, resulting in alterations in the activity of each enzyme: exon 1 in *DNASE1L3* and exon 1 in *DNASE2* (Fig 1). The specificity of the products amplified was evaluated by melting curve analysis for both *DNASE1L3* and *DNASE2* CNVs (S1 Fig). In the Q-PCR analysis, standard curves for each of the target CNV regions, together that for the single-copy *ZNF80* gene as a reference, were generated using a 2-fold serially diluted series of each chimeric vector as a template (Fig 2). For *DNASE* CNV analysis, the PCR efficiencies for the *ZNF80* and the target *DNASE1L3* CNV were 96.6±1.95% (slope coefficient, -3.41±0.0488) and 97.1±2.95% (slope coefficient, -3.39±0.0759), respectively, while those for the *ZNF80* and the target *DNASE2* CNV were 97.5±1.87% (slope coefficient, -3.38±0.0472) and 97.5±2.39% (slope coefficient, -3.39±0.0615), respectively. Since the quantitative accuracy of Q-PCR analysis depends on proper normalization, these results demonstrated that copy number estimation using this method was reliable. Furthermore, in order to confirm the validity of copy number determination using this newly developed CNV analysis method, simulated analysis of the *DNASE1L3* CNV copy number was performed using mixtures of three vectors–pCRII/DN1L3, pCRII/ZNF or DN1L3-ZNF–in varying proportions as a template. All of the simulated assays yielded the expected number of copies (Fig 3), indicating the reliability of this method. Therefore, this simple, newly developed Q-PCR method for CNV analysis permits reliable determination of copy number in the CNVs located in these DNase genes.

We examined the distribution of the CNVs in each of the genes encoding DNase 1L3 and DNase II, separately, in Japanese and German populations using Q-PCR analysis of the target regions common to all of the CNVs that could potentially lead to alterations in the activity levels of the enzymes; the determined *DNASE1L3* and *DNASE2* CNVs diploid copy numbers are summarized in Table 2. In both populations, only 2 diploid copy numbers for all of the *DNASE* CNVs were distributed; no loss or gain of copy was evident in any of the *DNASE1L3* and *DNASE2* CNVs.

**Table 2:** Estimated diploid copy number† of the CNVs in each of the genes encoding DNase 1L3 and DNase II in Japanese and German populations.

Gene	Estimated copy number					Copy number calculated (mean±SD)			
	1	2	3	4	Total	1	2	3	4
					Japanese				
*DNASE1L3*	0	265	0	0	265	NA	1.83±0.12	NA	NA
*DNASE2*	0	265	0	0	265	NA	1.90±0.12	NA	NA
					German				
*DNASE1L3*	0	80	0	0	80	NA	1.77±0.09	NA	NA
*DNASE2*	0	80	0	0	80	NA	1.92±0.10	NA	NA

† The diploid copy numbers of the CNV in each of the DNase genes were estimated based on the copy number calculated using the following

formula: (amount of *DNASE* amplicons)/(amount of *ZNF80* amplicons) x 2.

NA: not applicable

## Discussion

Several CNVs have been found in the genes encoding DNases 1L3 and II which are implicated in autoimmune diseases. In the present study, we focused on the target region common to all of the CNVs in *DNASE1L3*, together with that for CNV in *DNASE2*, likely giving rise to alterations in the *in vivo* levels of enzyme activity through loss or gain of copy in the CNVs (Fig 1). It was anticipated that this would allow evaluation of the distribution of the relevant CNVs in the genes, thus clarifying the overall effect of the CNVs on *in vivo* enzyme activities. For the CNV analysis, a simple and reliable newly developed Q-PCR method was employed (Figs 2 and 3, and S1 Fig). To our knowledge, the present study is the first to have comprehensively clarified the distribution of the CNVs in the autoimmune-related DNase genes, *DNASE1L3* and *DNASE2*.

Among all the CNVs examined in these DNase genes, those resulting in loss of copy would be expect to cause a reduction of the *in vivo* level of the corresponding enzyme activity. DNase 1L3 present in serum was assumed to be concerned with DNase I during DNA degradation, providing effective clearance after exposure or release from dying cells [19,20]. Low levels of serum DNase 1L3 activity have been demonstrated in patients with SLE[21]. Furthermore, the failure of efficient DNA clearance due to loss/reduction of DNase II function, being directly implicated in engulfment-mediated DNA degradation, would result in autoimmune dysfunction [22,23]. On the other hand, homozygous Caucasian carriers of the SNP rs12609744 *G* allele, the rs11085823 *G* allele, and the rs7249143 *A* allele in the 5’-upstream region of *DNASE2*, leading to low *in vivo* levels of enzyme activity through reduction of promoter activity in the gene, are considered to have an increased risk of rheumatoid arthritis [24,25]. These previous findings imply that SNVs/SNPs or Indels in the genes encoding *DNASE1L3*, and *DNASE2*, resulting in variants responsible for enzymes that are inactive or show low activity, might be substantially responsible for the genetic backgrounds implicated in the etiology and pathophysiological conditions of autoimmune diseases. However, the CNV analysis of autoimmune-related DNase genes in the present study demonstrated that only the 2 diploid copy number was present in the CNVs found in Japanese and German populations (Table 2). Notably, it was found that loss of the copy giving rise to abolishment of the activity in all of the autoimmune-related DNase genes was not widely distributed.

The distribution of gain/loss of copy in each of the CNVs examined in *DNASE1L3* and *DNASE2* released in the Database of Genomic Variants is summarized in S1 Table. Among them, 1 gain of copy and 1 loss of copy were observed out of sample size of 95 in nsv634703 in *DNASE1L3* and nsv833754 in *DNASE2*, respectively. Although this distribution in both of the CNVs would suggest about 3 instances of gain/loss of copy, at least in the present Japanese study population (*n* = 265), no gain/loss of copy was actually observed. Considering these findings, it seems plausible to conclude that, with regard to the CNVs resulting in alterations of *in vivo* DNase activity, these human autoimmune-related DNase genes show low genetic diversity, at least in the present Japanese population.

A large number of SNVs/SNPs in *DNASE1L3*, and *DNASEII* have been registered in the NCBI dbSNP database. We have continued a series of comprehensive investigations on the effects of the non-synonymous SNVs/SNPs in these DNase genes on the catalytic activities of the respective enzymes, thereby evaluating the functionality of each SNP [7,25–28]. Based on our expression analysis of the recombinant DNases, 5 and 4 SNPs producing loss-of-function variants of the enzymes encoded by *DNASE1L3* and *DNASE2*, respectively, were subsequently identified. However, all of these functional SNVs/SNPs resulting in loss of function exhibited a monoallelic homozygote of the wild allele in the study populations including a Japanese population, showing low heterogeneity comparable to the CNVs. Thus, it is plausible to assume that *DNASE1L3*, and *DNASE2* have been well conserved at the activity level during the evolution of human populations, thereby avoiding any marked reduction of the respective DNase activities. On the other hand, loss-of-function mutation of the DNase 1L3 gene has been identified in SLE patients [8]. Recently, Sisirak *et al*. [29] demonstrated that DNASE1L3-deficient mice rapidly develop autoantibodies to DNA and chromatin, followed by an SLE-like disease. Furthermore, knockout of DNase II has been reported to lead to rheumatoid arthritis-like conditions in mice [10]. These facts imply that both the CNVs and functional SNVs leading to abolishment of the DNase activity, irrespective of the extent of their distribution, in the genes encoding these autoimmune-related DNases might be considered genetic factors that are implicated in autoimmune dysfunction. Obviously, in order to confirm any clinical association of these CNVs and functional SNVs in *DNASE1L3*, and *DNASE2* with the incidence of autoimmune diseases, their distribution in various patient groups will need to be examined.

## Supporting information

S1 Fig Melting curve analysis results for (**A**) 81-bp amplicon for *DNASE1L3* CNVs and (**B**) 120-bp amplicon for *DNASE2* CNV.
(JPG)

S1 Table Distribution† of targeted CNVs in *DNASE1L3* and *DNASE2*. (DOCX)
