# Supplementary figures and images for: Low genetic heterogeneity of copy number variations (CNVs) in the genes encoding the human deoxyribonucleases 1-like 3 and II potentially relevant to autoimmunity
Source: PLoS One. 2019 Apr 25;14(4):e0215479. doi: 10.1371/journal.pone.0215479 (PMC6483174; doi:10.1371/journal.pone.0215479)

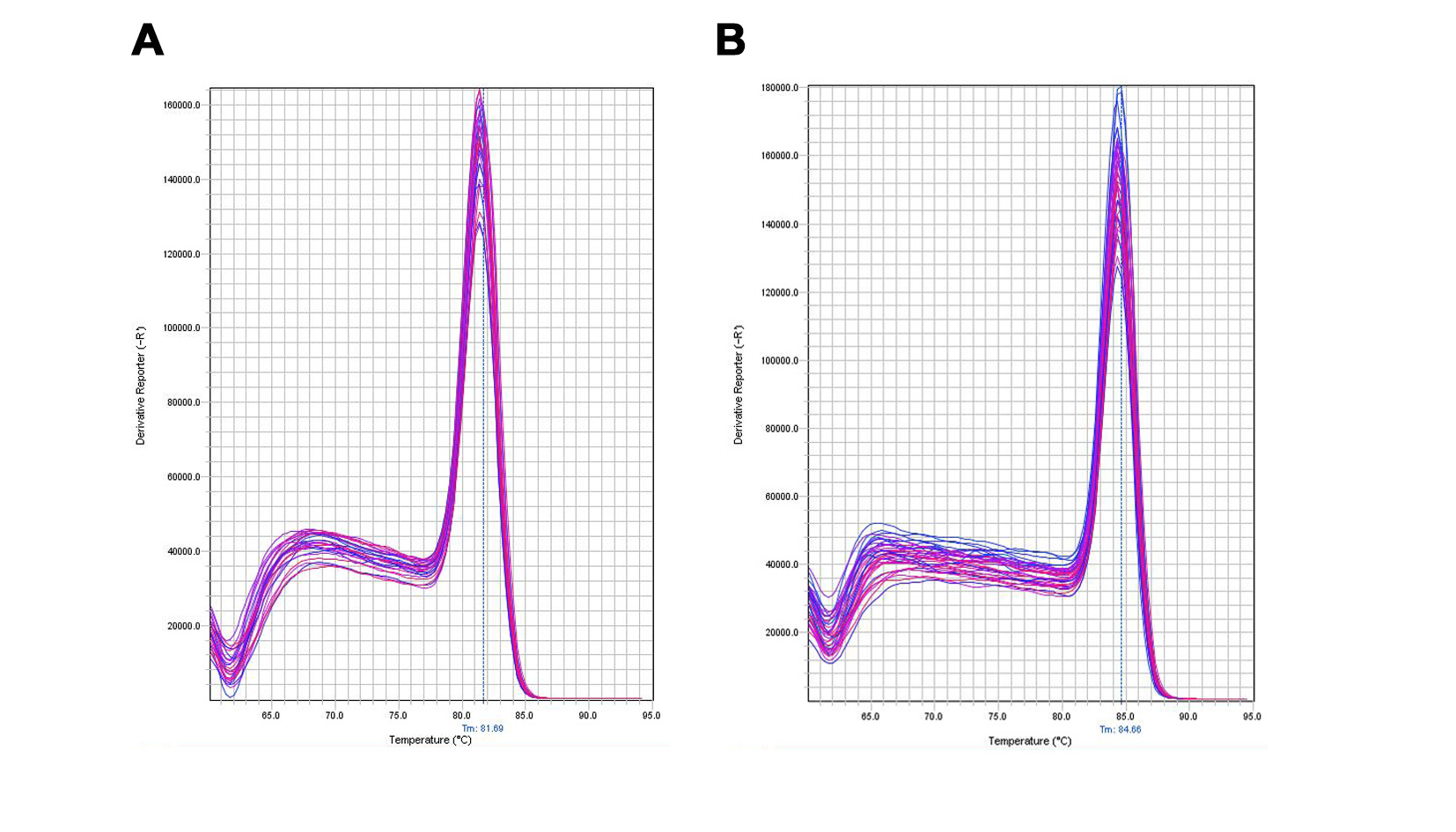

Supplement: S1 Fig — Melting curve analysis results for (A) 81-bp amplicon for DNASE1L3 CNVs and (B) 120-bp amplicon for DNASE2 CNV. (JPG) [file pone.0215479.s001.jpg]
