# Supplementary material for: Low genetic heterogeneity of copy number variations (CNVs) in the genes encoding the human deoxyribonucleases 1-like 3 and II potentially relevant to autoimmunity
Source: PLoS One. 2019 Apr 25;14(4):e0215479. doi: 10.1371/journal.pone.0215479 (PMC6483174; doi:10.1371/journal.pone.0215479)
Supplement: S1 Table — (DOCX) [file pone.0215479.s002.docx]

**Supplementary table 1**. Distribution† of targeted CNVs in *DNASE1L3* and *DNASE2*

CNV Sample size Observed gain Observed loss

*DNASE1L3*

nsv528147 2026 1 0

nsv634703 95 1 0

nsv1001814 29084 1 0

esv3568767 873 0 1

*DNASE2*

nsv833754 95 0 1

†Taken from the Database of Genomic Variants (Feb. 2019).
